# Mathematical modeling of pharmacokinetics and pharmacodynamics of losartan in relation to *CYP2C9* allele variants

**DOI:** 10.3389/fsysb.2025.1504077

**Published:** 2025-05-21

**Authors:** Dmitry Babaev, Elena Kutumova, Fedor Kolpakov

**Affiliations:** ^1^ Department of Computational Biology, Sirius University of Science and Technology, Sirius, Krasnodar Region, Russia; ^2^ Laboratory of Bioinformatics, Federal Research Center for Information and Computational Technologies, Novosibirsk, Russia

**Keywords:** arterial hypertension, losartan, pharmacokinetics, pharmacodynamics, CYP2C9, mathematical modeling, BioUML, personalized medicine

## Abstract

Losartan is a selective angiotensin II AT1-receptor antagonist for the treatment of arterial hypertension and heart failure. It is converted to a pharmacologically active metabolite carboxylosartan (E-3174) in the liver mainly by CYP2C9 enzyme, a member of the cytochrome P450 superfamily. The gene encoding this protein is highly polymorphic: numerous single nucleotide polymorphisms that alter the enzyme function have been described in the literature. The most widespread *CYP2C9* alleles are CYP2C9*1 (wild-type), CYP2C9*2, and CYP2C9*3. Here we performed mathematical modeling of the metabolism of orally administered losartan to E-3174 taking into account combinations of the most common *CYP2C9* alleles. Next, using the previously created model of the human cardiovascular and renal systems, we demonstrated that the blood pressure response to losartan therapy in a cohort of virtual hypertensive patients depended on *CYP2C9* allelic variants. Individuals with the CYP2C9*1/CYP2C9*1 genotype responded better to treatment than patients carrying CYP2C9*2 or CYP2C9*3 alleles. The results of the modeling can potentially be used for personalization of drug therapy for arterial hypertension.

## 1 Introduction

Cytochrome P450 (CYP) is a superfamily of enzymes that catalyze oxidative biotransformation of many drugs and other lipophilic compounds (Nelson, 2006). Most of the genes of this superfamily in humans are grouped into 18 families and 44 subfamilies and perform specific endogenous functions, including the biosynthesis of steroid hormones, prostaglandins, bile acids and other compounds (Nebert and Russell, 2002). However, only some of these genes (from the CYP1, 2, and 3 families) are involved in an oxidative drug metabolism. CYP2C is a subfamily of the CYP2 family that contains four genes: *CYP2C8*, *CYP2C9*, *CYP2C18*, and *CYP2C19* (Takahashi and Echizen, 2001). Among them, *CYP2C9* is a major gene, which accounts for about 20% of total liver microsomal proteins and metabolizes approximately 13%–17% of all clinical drugs, including S-warfarin, phenytoin, tolbutamide, glipizide, glyburide, torsemide, losartan, etc. (Miners and Birkett, 1998; Zanger et al., 2008; Zanger and Schwab, 2013).

The *CYP2C9* gene, located on chromosome 10q24 (Gray et al., 1995, p. 2), spans about 55 kb with 9 exons (GenBank accession numbers: L16877 to L16883) and encodes a protein of 490 amino acid residues, weighing 55.5 KDa^1^. Many single nucleotide polymorphisms (SNPs) were identified in *CYP2C9*, making it highly polymorphic (Lee et al., 2002). To date, 85 allelic variants of *CYP2C9* with different catalityc activity have been annotated^2^.

**FIGURE 1:**
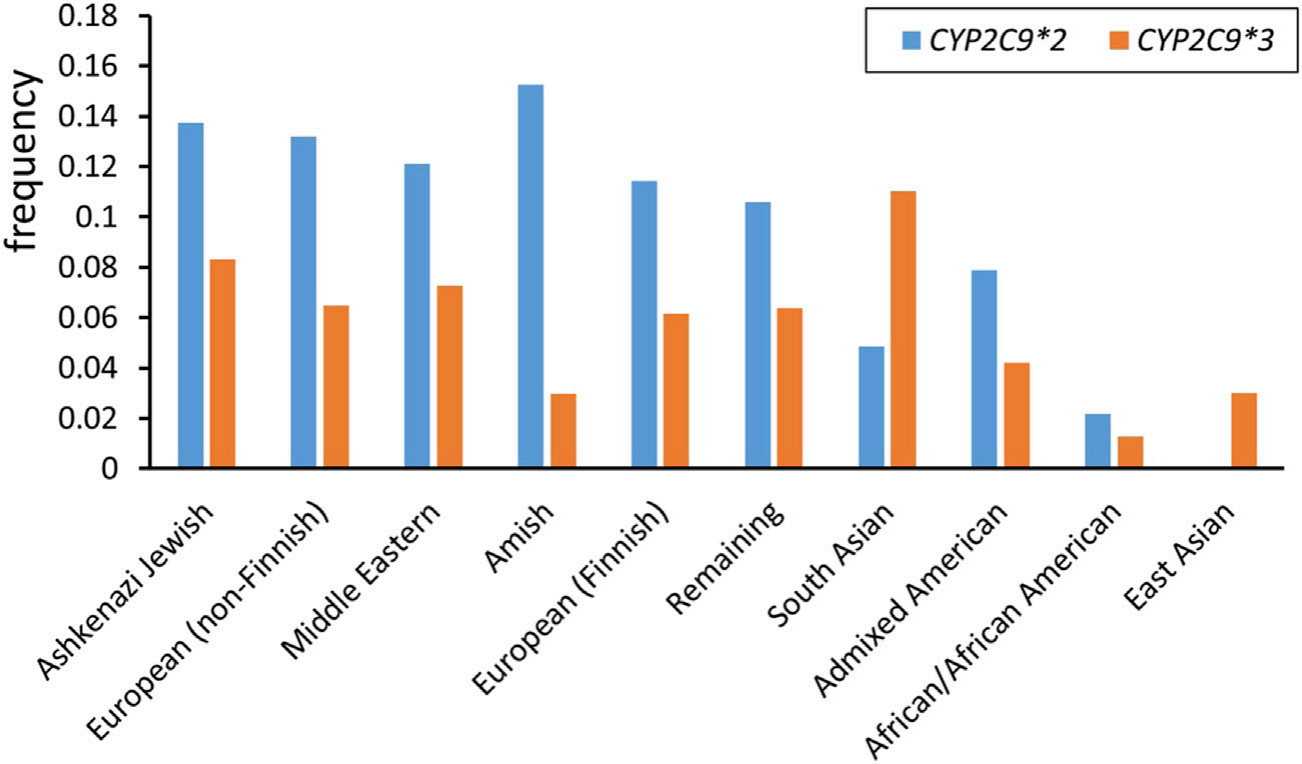
CYP2C9*2 and CYP2C9*3 allele frequencies among different ethnicities (data from gnomAD v4.1.0)^3^.

The wild-type *CYP2C9* allele is designated as CYP2C9*1. A cytosine to thymine transversion at nucleotide 430 encodes for an arginine to cysteine replacement at amino acid residue 144 (Arg144Cys), producing the CYP2C9*2 variant allele (Ged et al., 1988; Stubbins et al., 1996). CYP2C9*3 denotes a gene with an adenine to cytosine transversion in the seventh exon at 1,075 nucleotide (A1075C), encoding an isoleucine to leucine replacement at amino acid residue 359 (Ile359Leu) (Sullivan-Klose et al., 1996) (Table 1). These two alleles are the most common variants with a reduced activity and their frequency greatly varies among ethnic groups (Figure 1) (Table 2). Along with these two alleles, there are other alleles found in different populations. For example, about 2% of the Chinese population are carriers of the CYP2C9*13 allele (Si et al., 2004) while some rare *CYP2C9* alleles, such as CYP2C9*5, CYP2C9*6, CYP2C9*8, CYP2C9*9, and CYP2C9*11, are presented only in Africans (Zhou et al., 2017).

**TABLE 1:** Characteristics of CYP2C9*2 and CYP2C9*3 alleles.

Allele name	rsID	Nucleotide substitution	Position of the substitution	Amino acid substitution
CYP2C9*2	rs1799853	C430T	3 exon	Arg144Cys
CYP2C9*3	rs1057910	A1075C	7 exon	Ile359Leu

rsID (reference SNP, cluster ID), unique number of the single nucleotide polymorphism.

**TABLE 2:** CYP2C9*2 and CYP2C9*3 allele frequencies in different ethnicities.

Ethnicity	CYP2C9*2	CYP2C9*3
Ashkenazi Jewish	0.1375	0.0832
European (non-Finnish)	0.1319	0.0649
Middle Eastern	0.1212	0.0728
Amish	0.1524	0.0296
European (Finnish)	0.1142	0.0616
Remaining	0.1061	0.0639
South Asian	0.0485	0.1101
Admixed American	0.0787	0.0420
African/African American	0.0217	0.0126
East Asian	0.0002	0.0302

Data from gnomAD v4.1.0.

As mentioned above, losartan is one of the drugs that is metabolized by CYP2C9. However, *in vitro* data demonstrate that not only CYP2C9 but also CYP3A4 is involved in the biotransformation of this compound (McCrea et al., 1999; Stearns et al., 1995). Numerous clinical trials have been conducted to determine the contribution of both enzymes to losartan metabolism. CYP3A4 inhibitors such as itraconazole (Kaukonen et al., 1998), erythromycin (Williamson et al., 1998) and cimetidine (Goldberg et al., 1995) have been shown to have no significant effect on the pharmacokinetics of losartan *in vivo*. At the same time, bucolome (Kobayashi et al., 2008) and amodiaquine (Wennerholm et al., 2006), which have been reported to inhibit CYP2C9-mediated reactions, significantly affect the metabolism of losartan. Thus, the CYP2C9 is the major enzyme responsible for the oxidation of this drug.

Losartan is a selective angiotensin II type 1 (AT1) receptor antagonist used in the treatment of arterial hypertension (Timmermans et al., 1993). Losartan is converted to carboxylosartan (E-3174) via a carbonyl intermediate (E-3179) (Stearns et al., 1995; U et al., 2001; Yun et al., 1995). E-3174 is thought to be responsible for the main pharmacological effect, as it has 10–40 times greater AT1-receptor blocking activity than losartan and also has a longer half-life (Lo et al., 1995). Expression of CYP2C9*2 and CYP2C9*3 alleles has been shown to significantly alter the pharmacokinetics of losartan and E-3174 *in vitro* and *in vivo* (U et al., 2001; Yasar et al., 2002).

Previously, Eleni Karatza and Vangelis Karalis developed a four-compartment pharmacokinetic model that describes the disposition of losartan and E-3174 (Karatza and Karalis, 2020). The main objective of their work was to create a model describing the secondary maxima observed in the plasma concentration-time profiles of losartan and E-3174. Their model incorporates a sinusoidal equation which illustrates open-close cycles of gastric pyloric valve. However, the model does not include genetic factors, such as different CYP2C9 activity. Therefore, the primary aim of the present study was to modify this model not only to predict plasma concentration of losartan and its metabolite but also to consider the *CYP2C9* genotype of individual patients (Babaev et al., 2024).

The secondary aim of the study was to use the previously created mechanistic cardiorenal model (Kutumova et al., 2022; Kutumova et al., 2021) to examine the blood pressure response to losartan therapy depending on the *CYP2C9* genotype. Apart from physiological processes such as blood circulation and the cardiac cycle, neurohumoral regulation, blood-tissue oxygen exchange, the renin-angiotensin-aldosterone system (RAAS), renal microcirculation and sodium transport across the nephron, renal sympathetic nerve activity, and regulation of water-sodium balance, this model also incorporates the therapeutic effects of various antihypertensive agents, including the direct renin inhibitor aliskiren, the ACE inhibitor enalapril, the angiotensin II receptor blocker losartan, the β-blocker bisoprolol, the calcium channel blocker amlodipine, and the thiazide diuretic hydrochlorothiazide. The model has previously been used to study the association between *ACE I/D* genotypes and the blood pressure response to treatment with RAAS inhibitors, primarily enalapril (Kutumova et al., 2024). Here we applied this model to conduct *in silico* studies in a population of virtual hypertensive patients with different allelic variants of *CYP2C9*. Accumulating the results on modeling the influence of various genetic factors on the arterial pressure regulation may further allow us to study their cross-influence on the development and treatment of arterial hypertension.

## 2 Material and methods

### 2.1 Mathematical model of losartan metabolism

**FIGURE 2:**
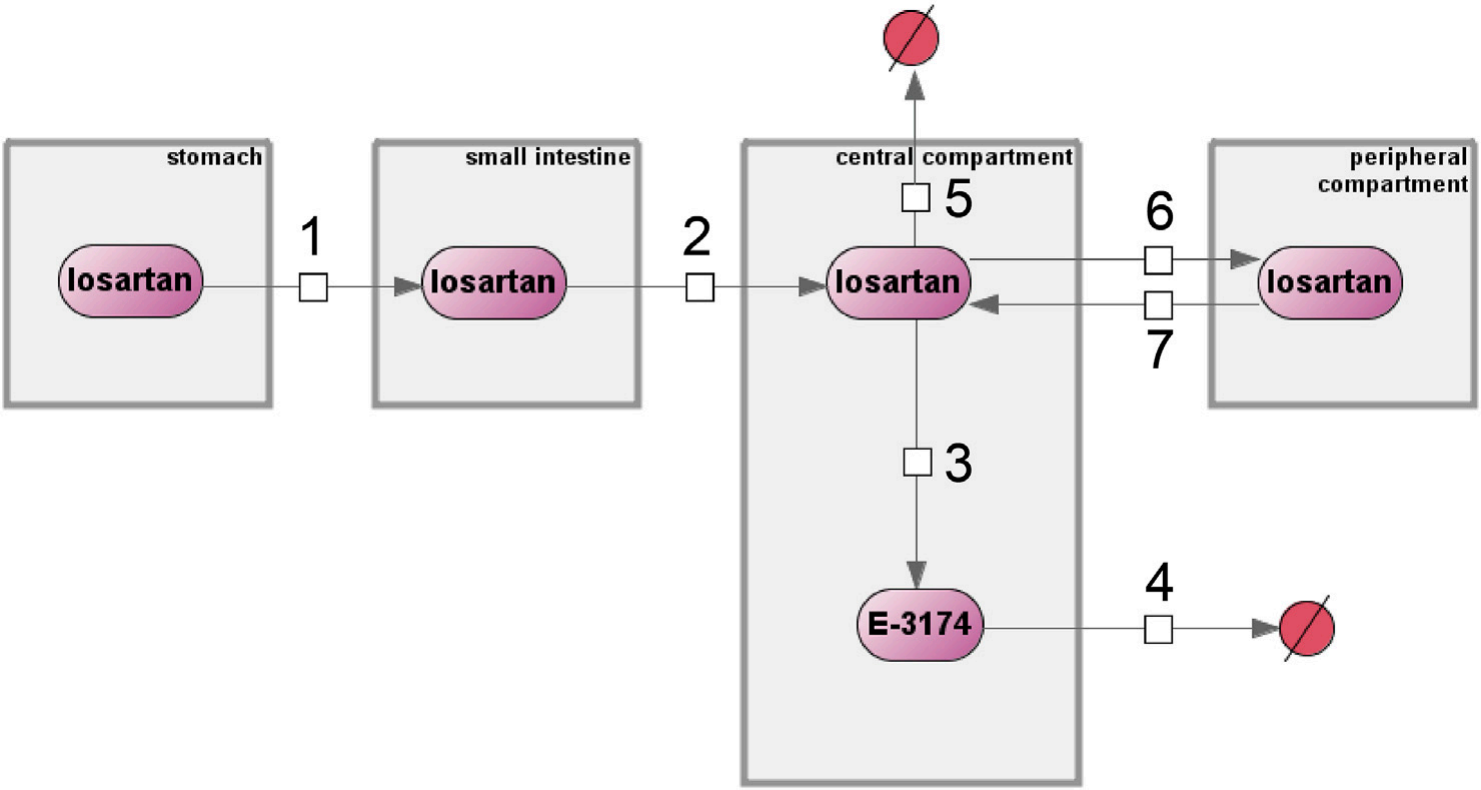
Original computational model of losartan metabolism by Eleni Karatza and Vangelis Karalis implemented in the BioUML software. Numbers 1 through 7 denote reactions, compartments are shown as gray rectangles, purple ovals represent chemical compounds, and excreted substances are shown as crossed-out red circles.

The original model (Karatza and Karalis, 2020) was reproduced in the BioUML^4^ software (Kolpakov et al., 2022; Kolpakov et al., 2019) using SBML format for technical representation (Keating et al., 2020) and SBGN format for visualization (Gambardella et al., 2009) (Figure 2).

The model includes four compartments: stomach, small intestine, central, and peripheral compartments. These compartments contain two substances, losartan and E-3174, which are interconnected through first-order reaction equations (numbers 1 through 7 in Figure 2). A detailed description of all equations and variables is given in Supplementary Tables S1, S2, respectively.

The model also includes discrete events, initial assignments, and algebraic and differential equations used to solve the model. The list of mathematical tools and variables can be found in Supplementary Tables S3, S4, respectively.

The pharmacokinetics of losartan were assessed by calculating the following parameters: area under the concentration-time curve (AUC) for losartan (AUC_losartan_) and its metabolite (AUC_E-3174_), AUC_losartan_ to AUC_E-3174_ ratio (AUC_ratio_), maximum plasma concentration (C_max_), time to reach C_max_ (t_max_), and terminal elimination half-life (t_1/2_) (Table 3).

**TABLE 3:** Pharmacokinetic parameters of the model and their description.

Pharmacokinetic parameter	Description
AUC	The area under the concentration-time curve (nmol∗h/L)
AUC_ratio_	AUC_losartan_ to AUC_E-3174_ ratio (unitless)
C_max_	The maximum plasma concentration (nM)
t_1/2_	The terminal elimination half-life (h)
t_max_	The time at which C_max_ occurred (h)

C_max_ and t_max_ were derived from the model simulation results. AUC_losartan_ and AUC_E-3174_ were calculated using the differential equations:

$$\frac{d\left({\text{AUC}}_{\text{losartan}}\right)}{d\left(time\right)}=C_{p},\frac{d\left({\text{AUC}}_{E-3174}\right)}{d\left(time\right)}=C_{m},$$

where *C_p_* and *C_m_* denote concentrations of losartan and E-3174, respectively.

Since the model was implemented using delay differential equations in the conversion of losartan to E-3174, the concentration of E-3174 in the first numerical steps of the simulation is zero. Consequently, the AUC_E-3174_ value is also zero. Therefore, a piecewise function was used to calculate AUC_ratio_:

$${\text{AUC}}_{\text{ratio}}=\left\{\begin{array}{l} 0,\text{if }C_{m}=0,\\ \frac{{\text{AUC}}_{\text{losartan}}}{{\text{AUC}}_{E-3174}},\text{otherwise} \end{array}\right.$$

The t_1/2_ value was calculated by linear regression analysis from the terminal linear part of the plasma concentration versus time semilogarithmic plot according to the following equation:

$${\text{t}}_{1/2}=\frac{\left({\text{t}}_{2}-{\text{t}}_{1}\right)\times \ln\left(2\right)}{\ln\left(\frac{{\text{C}}_{1}}{{\text{C}}_{2}}\right)}$$

where C_1_ and C_2_ are the concentrations of the corresponding substance at times t_1_ and t_2_, respectively, according to the linear regression line (Toutain and Bousquet-Mélou, 2004).

### 2.2 Mathematical model of the human cardiovascular and renal systems

To test the losartan metabolism model in a population of virtual patients with different *CYP2C9* genotypes, we used the computational model of the human cardiovascular and renal systems that, in particular, simulates the pharmacological effects of losartan on cardiovascular and renal parameters (Kutumova et al., 2022; Kutumova et al., 2021). The model is discrete-continuous and consists of a system of ordinary differential equations with several discrete events corresponding to instantaneous changes in the modeled dynamics (e.g., the transition from systole to diastole). This model is available in the BioModels database (Malik-Sheriff et al., 2020) with ID MODEL2202160001^5^.

A virtual patient is a single equilibrium parameterization of the model within physiological limits. In the current work, we used 100 virtual hypertensive patients from our previous study on antihypertensive therapy modeling (Kutumova et al., 2022).

### 2.3 Modeling the impact of *CYP2C9* genetic variants on losartan treatment response

To simulate treatment of virtual patients with losartan, we used the following equation from the cardiorenal model (Kutumova et al., 2022), which reduces the rate of angiotensin II binding to AT1-receptors:

$$ARB=k_{block}\times {Losartan}_{treatment},$$

where *ARB* (angiotensin receptor blocking effect) is the AT1-receptor blocking activity of the drug, *Losartan_treatment_* is a discrete parameter that can take values of 0 (no treatment) or 1 (treatment course), and *k*_*block*_ is the total AT1-receptor blocking activity of losartan across *CYP2C9* genotypes.

The value of the parameter *k*_*block*_ is fed into the cardiorenal model from the losartan metabolism model and depends on the *CYP2C9* allelic variant. Parameter values for the different *CYP2C9* genotypes were estimated using the following considerations. Losartan 25 mg orally once daily has not been shown to produce clinically significant reductions in blood pressure compared with placebo (Gradman et al., 1995). A similar effect in the cardiorenal model is given by *k*_*block*_ = 0.1. In addition, for daily doses of 50 and 100 mg losartan, *k*_*block*_ values were estimated to be 0.886 and 0.954 (Kutumova et al., 2022). Using these doses in the pharmacokinetic model with parameters fitted to the *CYP2C9∗1/CYP2C9∗1* genotype (wild-type), we calculated the corresponding AUC_E-3174_ values. Thus, we received three relationship points between *k*_*block*_ values and corresponding AUC_E-3174_ values (Supplementary Table S5).

To describe this dependence, we used the following E-max model (Kirby et al., 2011):

$$k_{block}=\frac{E_{\max}\times \text{AU}{C_{E-3174}}^{\alpha }}{E{D_{50}}^{\alpha }+\text{AU}{C_{E-3174}}^{\alpha }}$$

The coefficients of the fitted E-max model are presented in Supplementary Table S6. Finally, using this equation, we extended the relationship between *k*_*block*_ and AUC_E-3174_ values to any values of AUC_E-3174_, i.e., for any *CYP2C9* allelic variant (Table 4) (Supplementary Figure S1).

**TABLE 4:** Correspondence between AUC_E-3174_ and *k*_*block*_ values for *CYP2C9* genotypes.

Genotype	AUC_E-3174_ (nmol*h/L)	*k*_*block*_ (unitless)
CYP2C9*1/CYP2C9*1	3996.6	0.886
CYP2C9*2/CYP2C9*2	3640.5	0.833
CYP2C9*3/CYP2C9*3	247.9	0.000
CYP2C9*1/CYP2C9*2	3833.3	0.866
CYP2C9*1/CYP2C9*3	3003.0	0.620
CYP2C9*2/CYP2C9*3	2628.8	0.410

### 2.4 Numerical solution of the models

To simulate the models, we used a version of the CVODE solver (Hindmarsh et al., 2004) ported to Java and adapted to the BioUML software interface.

CVODE solves initial-value problems for systems of ordinary differential equations in real N-space. It is used to solve both stiff and non-stiff systems by variable-order and variable-step multistep methods. CVODE contains two groups of multistep formulas that are suitable for different systems. For non-stiff problems, CVODE includes the Adams-Moulton formulas, and for stiff problems, the backward differentiation formulas. The coefficients for these methods are determined based on the method type, its order, the recent history of step sizes, and the normalization α_n,0_ = −1.

### 2.5 Parameter estimation

To solve the inverse problem of identifying model parameters based on experimentally measured variables, nonlinear optimization methods were used.

This problem was solved by finding the minimum of the objective function, which was calculated by summing the squares of the distances from the experimental points to the points predicted by the model. If the problem required additional constraints imposed on some variables of the model, a penalty function was used to assess their feasibility.

BioUML contains the following optimization methods:

1. Stochastic Ranking Evolutionary Strategy (SRES) (Runarsson et al., 2000),
2. DIRECT algorithm (Jones, 2009),
3. Quadratic Hill-climbing (Goldfeld et al., 1966),
4. Multi-objective particle swarm optimization (MOPSO) (Sierra and Coello, 2005),
5. Multi-objective cellular genetic algorithm (MOCell) (Nebro et al., 2009),
6. Adaptive simulated annealing (ASA) (Ingber, 1996).

Three of these methods (SRES, MOPSO and MOCell) were most suitable for our task. We compared the optimization results of these methods for our problem and found that they differed by no more than 5%. Therefore, we arbitrarily selected one of them (SRES) for further calculations.

### 2.6 Parameter identifiability

After estimating the model parameters based on experimental data, it is important to understand how accurately these parameters have been estimated in terms of the quantity and quality of the data. This understanding is necessary for further investigation of model predictions and can be provided by analyzing the parameters for identifiability (Raue et al., 2010; Raue et al., 2009). To study the sensitivity of the objective function to changes in a fitting parameter, we exclude it from the optimization process with a fixed value that gradually increases and then decreases compared to the optimal solution. In this way, we determine the influence of this parameter on the value of the objective function (i.e., the quality of the experimental data approximation). If the shift of the parameter in any direction along the numerical axis leads to a significant increase in the objective function, then it is identifiable. If a significant increase in the objective function occurs when moving in only one direction, then the parameter is partially identifiable. Otherwise, it is impossible to determine the parameter based on the available experimental data, that is, it is unidentifiable.

### 2.7 Digitizing of plots

To train the losartan metabolism model, we needed experimental plasma concentrations of the drug and its active metabolite in individuals with homozygous *CYP2C9* genotypes (CYP2C9*1/CYP2C9*1, CYP2C9*2/CYP2C9*2, and CYP2C9*3/CYP2C9*3). We were able to find only one article with the time courses of these concentrations after a single oral dose of 50 mg losartan potassium for all the above genotypes (Yasar et al., 2002).

To digitize this data from the figures in the original study, we used Plotdigitizer^6^ software. In analyzing the data, we decided not to use the first losartan point of the concentration-time curve from the CYP2C9*2/CYP2C9*2 plot because immediately after drug administration (time = 0), the concentration of losartan was 860 nM, which means that the absorption of the drug occurred instantly and is unlikely since it takes some time to get from the stomach to the small intestine and then into the blood. In addition, the maximum concentration values of losartan and E-3174 in the figures were replaced with the tabulated values of C_max_ for the corresponding genotypes from the same study to obtain more plausible results. The results of data digitizing are presented in Supplementary Table S7.

### 2.8 Software

To develop and analyze the model, we used the BioUML software (version 2023.3), a Java-based integrated environment for the modeling of different biological systems (Kolpakov et al., 2022; Kolpakov et al., 2019).

## 3 Results

### 3.1 Estimation of CYP2C9 activity for homozygous *CYP2C9* genotypes

The original model of losartan pharmacokinetics was validated using experimental data on the time-dependent plasma concentrations of losartan and its metabolite after a single oral dose of 100 mg losartan potassium (Figure 2) (Karatza and Karalis, 2020). At the same time, in the clinical study of individuals with different genotypes of *CYP2C9*, which plays a major role in the metabolism of the drug, a dosage of 50 mg was used (Yasar et al., 2002). Directly changing the drug dosage from 100 to 50 mg in the model showed a significant discrepancy between the experimental data and the model predictions (Supplementary Figure S2). Furthermore, the original model did not take into account the different CYP2C9 activities. Thus, to refine the model to incorporate *CYP2C9* genetic factors, it was necessary to recalibrate its parameters.

By analyzing the list of these parameters (Supplementary Table S2), we came to the conclusion that the value of the parameter *b* in the sinusoidal equation (number 1 in Figure 2), which describes the periodic opening and closing of the pyloric valve of the stomach (Supplementary Table S1), has a physiological justification. It consists in the fact that the cycle of the migrating motor complex (a regular pattern of gastric motility during fasting) (Deloose et al., 2012) varies on average from 85 to 115 min (Feher, 2017), and at *b* = 3.95 (the value in the original model), the resulting period falls within this time interval.

In addition, the parameter *k*_*m*_ from reaction 3 in Figure 2 is a first-order kinetic constant characterizing the rate of conversion of losartan to E-3174, i.e., the activity of CYP2C9*.* Therefore, we estimated the unique value of this parameter for each of the three homozygous genotypes.

The final *k*_*m*_ values for each genotype are presented below:

$$k_{m}\left(CYP2C9*1/CYP2C9*1\right)=1.056\;h^{-1},$$

$$k_{m}\left(CYP2C9*2/CYP2C9*2\right)=0.823\;h^{-1},$$

$$k_{m}\left(CYP2C9*3/CYP2C9*3\right)=0.025\;h^{-1}.$$

The optimized values of all other parameters are listed in Supplementary Table S8.

**FIGURE 3:**
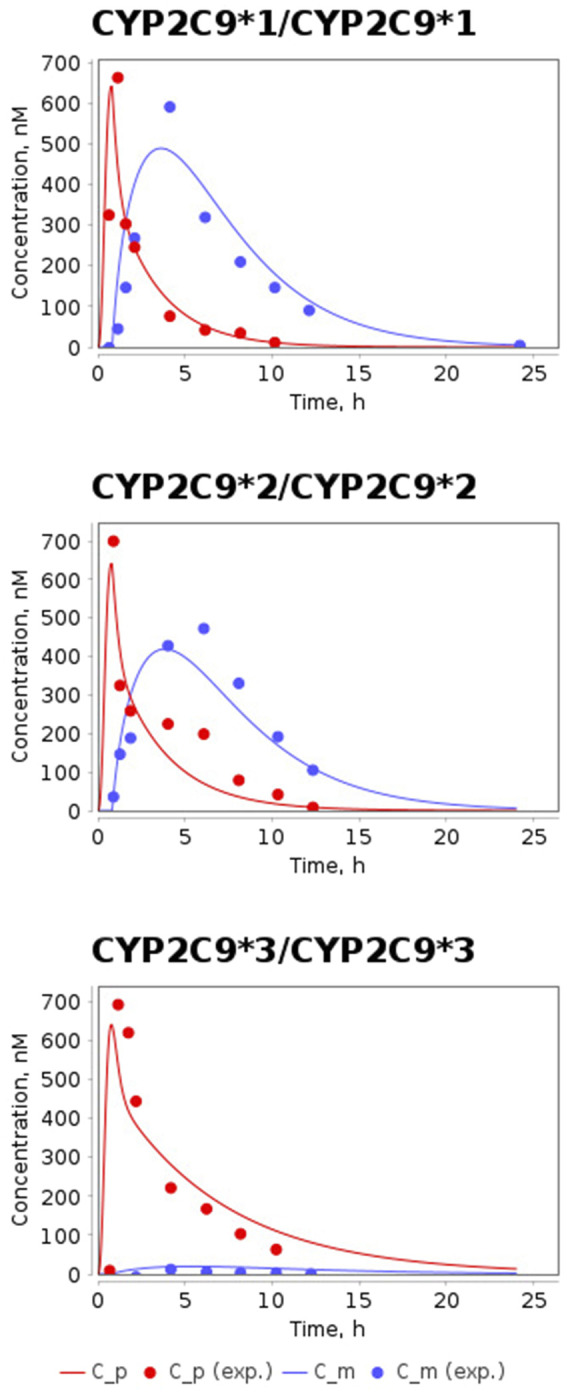
Model prediction of losartan and E-3174 plasma concentrations for three *CYP2C9* homozygous genotypes after a single 50 mg oral dose of losartan potassium. C_p, predicted profile of losartan; C_m, predicted profile of E-3174; C_p (exp.), experimental time-course of losartan; C_m (exp.), experimental time-course of E-3174. In experimental work (Yasar et al., 2002) 6 patients were included in CYP2C9*1/CYP2C9*1 group, 3 patients - in CYP2C9*2/CYP2C9*2 group, and only one patient - in CYP2C9*3/CYP2C9*3 group.

**FIGURE 4:**
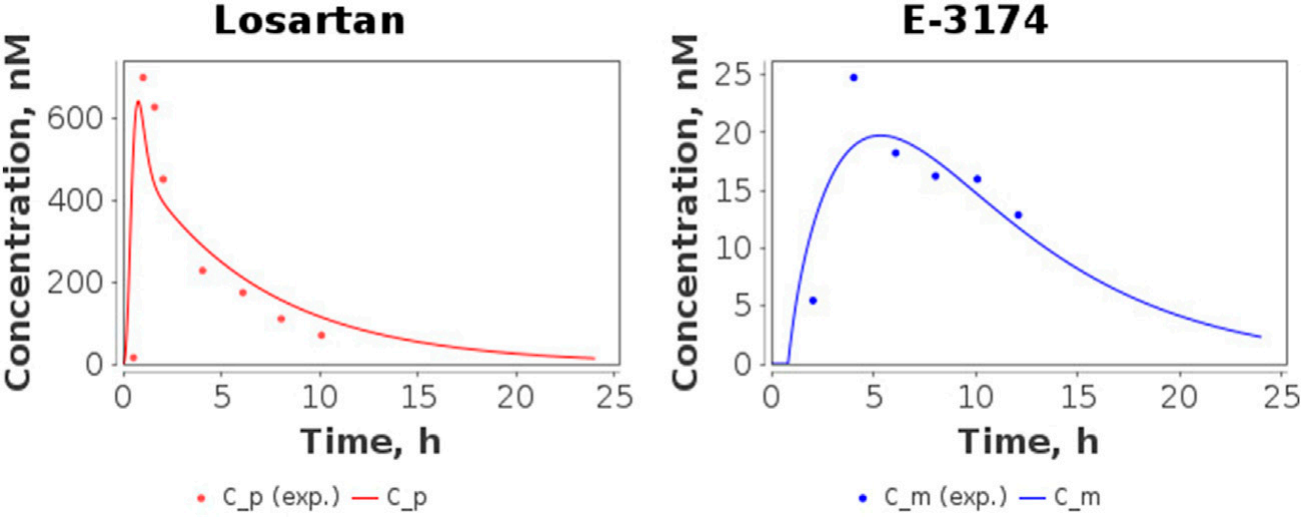
Model predictions of plasma concentrations of losartan and E-3174 for the CYP2C9*3/CYP2C9*3 genotype after a single 50 mg oral dose of losartan potassium. C_p, predicted losartan profile; C_m, predicted E-3174 profile; C_p (exp.), experimental time-course of losartan; C_m (exp.), experimental time-course of E-3174. In the experimental study (Yasar et al., 2002), only one patient was included in this group.

The time-dependent concentrations of losartan and E-3174 after a single oral dose of 50 mg losartan potassium for three *CYP2C9* homozygotes predicted by the model after parameter redefinition, as well as experimentally obtained data for individuals with the same genotypes (Yasar et al., 2002), are shown in Figure 3. Because the concentration of losartan was significantly higher than the concentration of its metabolite for CYP2C9*3/CYP2C9*3 genotype, we plotted profiles of both substances separately (Figure 4). The predicted curves accurately describe the clinical data.

After validation of the model, we calculated the values of the pharmacokinetic parameters (AUC, AUC_ratio_, C_max_, t_1/2_, and t_max_) (Table 3) for the simulated profiles of losartan and E-3174 in homozygous genotypes and compared them with the corresponding values calculated from the experimental data (Table 5).

**TABLE 5:** Comparison of key pharmacokinetic parameters for *CYP2C9* homozygous genotypes.

Genotype	Pharmacokinetic parameter	Clinical data: mean ± SD, range	Model prediction
CYP2C9*1/CYP2C9*1	C_max, losartan_ (nM)	675 ± 417, 328–1,404	641.5
	t_max, losartan_ (h)	0.9 ± 0.4	0.8
	t_1/2, losartan_ (h)	1.9 ± 0.6, 1.2–2.9	1.8
	AUC_losartan_ (nmol*h/L)	1,697 ± 1,061, 522–3,373	1393.9
	C_max, E-3174_ (nM)	603 ± 443, 282–1,451	488.8
	t_max, E-3174_ (h)	3.9 ± 1.9	3.6
	t_1/2, E-3174_ (h)	4.0 ± 1.1, 2.7–5.9	2.7^a^
	AUC_E-3174_ (nmol*h/L)	4,346 ± 2,584, 2,162–9,183	3996.6
	AUC_ratio_ (unitless)	0.3 ± 0.1, 0.2–0.5	0.3
CYP2C9*2/CYP2C9*2	C_max, losartan_ (nM)	713 ± 423, 448–1,201	641.5
	t_max, losartan_ (h)	0.6 ± 0.3	0.8
	t_1/2, losartan_ (h)	2.0 ± 0.6, 1.3–2.5	2.1
	AUC_losartan_ (nmol*h/L)	1912 ± 438, 1,419–2254	1584.0
	C_max, E-3174_ (nM)	486 ± 210, 256–669	419.5
	t_max, E-3174_ (h)	5.8 ± 2.9	3.8
	t_1/2, E-3174_ (h)	3.8 ± 1.2, 2.9–5.2	2.8
	AUC_E-3174_ (nmol*h/L)	4,104 ± 2,097, 1,931–6,116	3640.5
	AUC_ratio_ (unitless)	0.6 ± 0.5, 0.33–1.2	0.4
CYP2C9*3/CYP2C9*3	C_max, losartan_ (nM)	706 ± 353	641.5
	t_max, losartan_ (h)	0.9 ± 0.4	0.8
	t_1/2, losartan_ (h)	3.6 ± 1.8	4.5
	AUC_losartan_ (nmol*h/L)	2,769 ± 1,385	3338.5
	C_max, E-3174_ (nM)	25 ± 12	19.7
	t_max, E-3174_ (h)	3.9 ± 1.9	5.4
	t_1/2, E-3174_ (h)	6.8 ± 3.4	5.1
	AUC_E-3174_ (nmol*h/L)	312 ± 156	247.9
	AUC_ratio_ (unitless)	8.9 ± 4.5	13.5^b^

C_max, losartan_, the maximum plasma concentration of losartan; C_max, E-3174_, the maximum plasma concentration of E-3174; t_max, losartan_, the time to reach C_max, losartan_; t_max, E-3174_, the time to reach C_max, E-3174_; t_1/2, losartan_, the apparent terminal elimination half-life of losartan; t_1/2, E-3174_, the apparent terminal elimination half-life of E-3174; AUC_losartan_, the area under the concentration-time curve of losartan; AUC_E-3174_, the area under the concentration-time curve of E-3174; AUC_ratio_, AUC_losartan_ to AUC_E-3174_ ratio.

In experimental work (Yasar et al., 2002) 6 patients were included in CYP2C9*1/CYP2C9*1 group, 3 patients - in CYP2C9*2/CYP2C9*2 group, and only 1 patient - in CYP2C9*3/CYP2C9*3 group.

^a^ The predicted value of the parameter does not fall within the mean ± SD experimental range, but falls within min - max experimental range.

^b^ The predicted value of the parameter does not fall within the min - max experimental range.

The values of all simulated pharmacokinetic parameters fell within the corresponding experimental range (Table 5). However, the t_1/2, E-3174_ value for the CYP2C9*1/CYP2C9*1 case was at the lower bound of the range. t_max_ values for losartan and E-3174 for different genotypes were not provided in the clinical study (Yasar et al., 2002). Therefore, we derived these values directly from the losartan and E-3174 concentration-time profiles reported in the article for homozygous *CYP2C9* genotypes. Note that we were only able to derive mean values for t_max_ from these data, while the variation among patients was unknown because blood samples for analysis were taken at fixed times (0, 0.5, 1, 1.5, 2, 4, 6, 8, 10, 12, 24 h). In addition, the group of patients with the CYP2C9*3/CYP2C9*3 genotype included only one individual (Yasar et al., 2002) and direct calculation of SD for all pharmacokinetic parameters in this case was also not possible. For the comparative analysis of experimental and simulated values, we assumed that the unknown SD values mentioned above could be the same as for other parameters in the CYP2C9*1/CYP2C9*1 group (SD varies from 28% to 73% for different parameters, i.e., is 50% on average). Considering SD = 50% for all parameters in CYP2C9*3/CYP2C9*3 and for t_max_ parameters in all groups, we found that all model-predicted values, except AUC_ratio_ for the CYP2C9*3/CYP2C9*3 case, fell within the mean ± SD range, while the simulated value AUC_ratio_ = 13.5 deviated from the experimental value of 8.9 by 51%.

### 3.2 Identifiability of the model parameters

**FIGURE 5:**
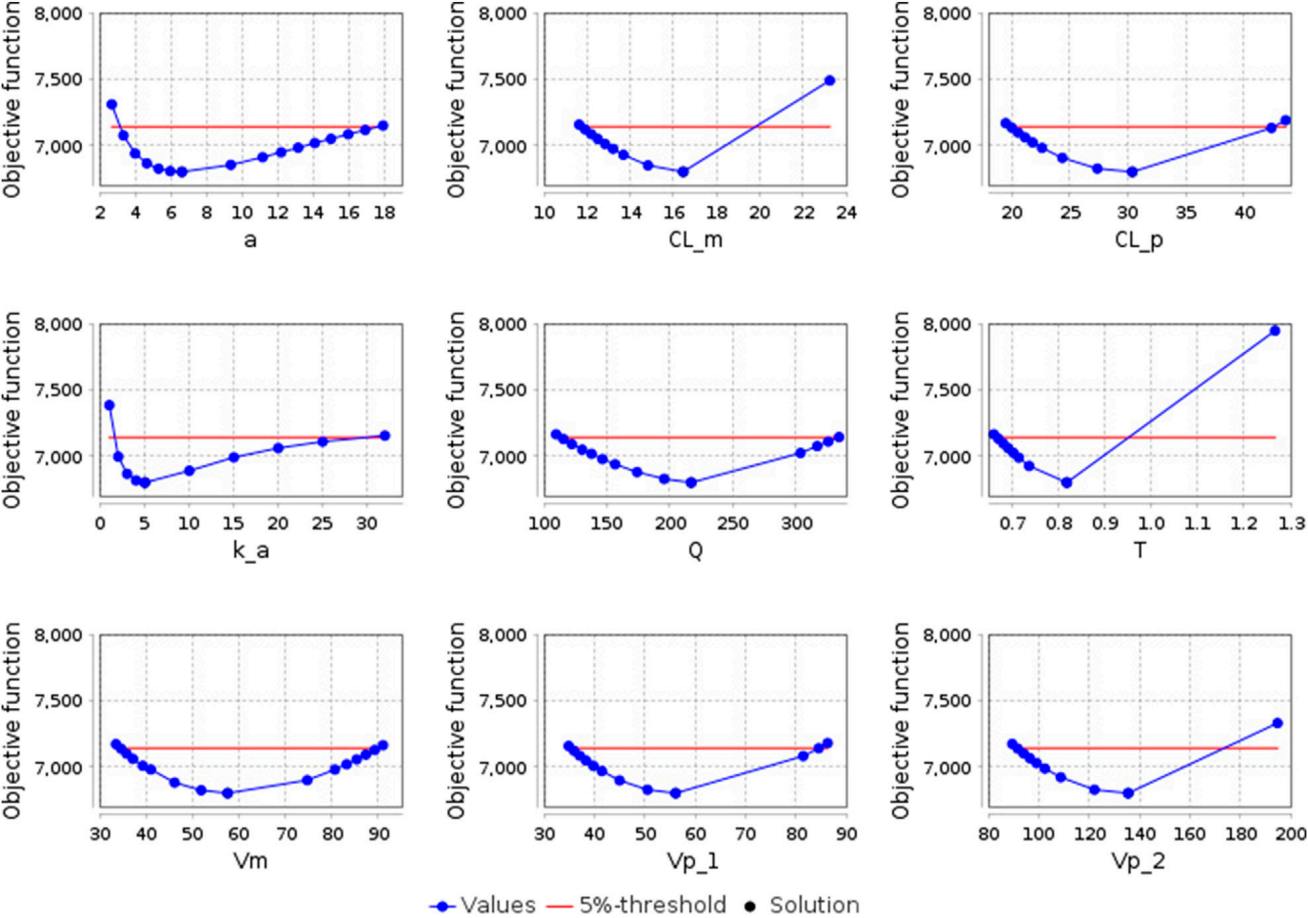
Identifiability plots of the model parameters: a - the amplitude of the sinusoidal equation, which describes open-close cycles of the gastric pyloric valve (h^-1^); CL_m - apparent clearance of E-3174 (L/h); CL_p - apparent clearance of losartan (L/h); k_a - rate constant of losartan absorption from the small intestine into the blood (h^-1^); Q - apparent inter-compartmental (central-peripheral) clearance of losartan (L/h); T - time delay in the conversion of losartan to E-3174 (h); Vm - apparent volume of distribution of E-3174 in the central compartment (L); Vp_1 - apparent volume of distribution of losartan in the central compartment (L); Vp_2 - apparent volume of distribution of losartan in the peripheral compartment (L). The blue dots represent the objective function values, the black dot indicates the optimal solution (coincides with the solution at the zero step of optimization), and the red line denotes the boundary value of the objective function (5% of the initial objective function value).

After optimizing the model parameters, we checked them for identifiability to ensure that the resulting solution is unique. As can be seen from Figure 5, all model parameters are identifiable.

### 3.3 Verification of the model

The model was tested using the data from the same experimental study (Yasar et al., 2002), but obtained for patients with heterozygous *CYP2C9* genotypes (CYP2C9*1/CYP2C9*2, CYP2C9*1/CYP2C9*3, and CYP2C9*2/CYP2C9*3).

Since the rate of conversion of losartan to E-3174 in the model was described using a first-order reaction equation, the arithmetic mean of the *k*_*m*_ values for the two homozygotes whose alleles were included in the heterozygote was used as *k*_*m*_ for this heterozygote:

$$\begin{array}{l} k_{m}\left(CYP2C9*1/CYP2C9*2\right)\\ =\frac{\left(1.056+0.823\right)}{2}=0.940\;h^{-1}, \end{array}$$

$$\begin{array}{l} k_{m}\left(CYP2C9*1/CYP2C9*3\right)\\ =\frac{\left(1.056+0.025\right)}{2}=0.541\;h^{-1}, \end{array}$$

$$\begin{array}{l} k_{m}\left(CYP2C9*2/CYP2C9*3\right)\\ =\frac{\left(0.823+0.025\right)}{2}=0.424\;h^{-1}. \end{array}$$

In the study by Yasar et al. (2002), there were no concentration-time curves for patients with heterozygous *CYP2C9* genotypes, so it was impossible to check how well the concentration-time curves of losartan and E-3174 simulated by the model matched the experimental ones (Supplementary Figure S3). Therefore, to verify the model, we compared pharmacokinetic parameter values from the clinical study Yasar et al., (2002) and those predicted by the model (Table 6).

**TABLE 6:** Comparison of key pharmacokinetic parameters for *CYP2C9* heterozygous genotypes.

Genotype	Pharmacokinetic parameter	Clinical data: mean ± SD, range	Model prediction
CYP2C9*1/CYP2C9*2	C_max, losartan_ (nM)	731 ± 489, 208–1,178	641.5
	t_max, losartan_ (h)	0.9 ± 0.4	0.8
	t_1/2, losartan_ (h)	2.1 ± 0.4, 1.6–2.5	1.9
	AUC_losartan_ (nmol*h/L)	1,521 ± 850, 750–2432	1,481.2
	C_max, E-3174_ (nM)	763 ± 565, 384–1,412	455.8
	t_max, E-3174_ (h)	3.9 ± 1.9	3.7
	t_1/2, E-3174_ (h)	4.3 ± 0.2, 4.1–4.3	2.7^b^
	AUC_E-3174_ (nmol*h/L)	5,564 ± 3,505, 3,355–9,605	3833.3
	AUC_ratio_ (unitless)	0.3 ± 0.1, 0.2–0.4	0.4
CYP2C9*1/CYP2C9*3	C_max, losartan_ (nM)	353 ± 160, 148–596	641.5^b^
	t_max, losartan_ (h)	0.9 ± 0.4	0.8
	t_1/2, losartan_ (h)	2.4 ± 0.5, 1.9–3.2	2.5
	AUC_losartan_ (nmol*h/L)	1,249 ± 248, 925–1,572	1921.7^b^
	C_max, E-3174_ (nM)	241 ± 102, 108–369	315.3
	t_max, E-3174_ (h)	3.9 ± 1.9	4.2
	t_1/2, E-3174_ (h)	5.6 ± 1.0, 4.5–7.0	3.1^b^
	AUC_E-3174_ (nmol*h/L)	2753 ± 898, 1,446–3740	3003.0
	AUC_ratio_ (unitless)	0.5 ± 0.2, 0.3–0.8	0.6
CYP2C9*2/CYP2C9*3	C_max, losartan_ (nM)	635 ± 388, 213–1,062	641.5
	t_max, losartan_ (h)	0.9 ± 0.4	0.8
	t_1/2, losartan_ (h)	3.0 ± 0.6, 2.2–3.5	2.8
	AUC_losartan_ (nmol*h/L)	2006 ± 632, 1,269–2639	2118.1
	C_max, E-3174_ (nM)	179 ± 39, 144–217	263.0^b^
	t_max, E-3174_ (h)	3.9 ± 1.9	4.4
	t_1/2, E-3174_ (h)	6.1 ± 1.6, 4.4–7.6	3.4^b^
	AUC_E-3174_ (nmol*h/L)	2134 ± 491, 1,749–2,849	2628.8^a^
	AUC_ratio_ (unitless)	0.9 ± 0.4, 0.5–1.3	0.8

C_max, losartan_, the maximum plasma concentration of losartan; C_max, E-3174_, the maximum plasma concentration of E-3174; t_max, losartan_, the time to reach C_max, losartan_; t_max, E-3174_, the time to reach C_max, E-3174_; t_1/2, losartan_, the apparent terminal elimination half-life of losartan; t_1/2, E-3174_, the apparent terminal elimination half-life of E-3174; AUC_losartan_, the area under the concentration-time curve of losartan; AUC_E-3174_, the area under the concentration-time curve of E-3174; AUC_ratio_, AUC_losartan_ to AUC_E-3174_ ratio.

In experimental work (Yasar et al., 2002) 3 patients were included in CYP2C9*1/CYP2C9*2 group, 5 patients - in CYP2C9*1/CYP2C9*3 group, and 4 patients - in CYP2C9*2/CYP2C9*3 group.

^a^ The predicted value of the parameter does not fall within the mean ± SD experimental range, but falls within min - max experimental range.

^b^ The predicted value of the parameter does not fall within the min - max experimental range.

Because concentration-time profiles of losartan and E-3174 were not available for heterozygous *CYP2C9* genotypes, we were unable to estimate t_max, losartan_ and t_max, E-3174_ values and instead used the corresponding values obtained for the CYP2C9*1/CYP2C9*1 case.

Table 6 demonstrates that the model predictions for heterozygous *CYP2C9* genotypes fit the experimental data worse than for homozygous genotypes. The simulated values of the following pharmacokinetic parameters are outside the experimental range: t_1/2, E-3174_ for the CYP2C9*1/CYP2C9*2 group, C_max, losartan_, AUC_losartan_ and t_1/2, E-3174_ for CYP2C9*1/CYP2C9*3, C_max, E-3174_ and t_1/2, E-3174_ for CYP2C9*2/CYP2C9*3. At the same time, the simulated AUC_E-3174_ value for the CYP2C9*2/CYP2C9*3 genotype is at the upper bound of the mean ± SD experimental range.

### 3.4 Simulation of losartan antihypertensive therapy

To assess the antihypertensive effect of losartan therapy in individuals with different *CYP2C9* genotypes, we used a previously developed cardiorenal model (Kutumova et al., 2022; Kutumova et al., 2021), which, in particular, reproduces the pharmacological action of losartan, and examined 100 virtual patients with arterial hypertension generated for it earlier (Kutumova et al., 2022). The distribution of their physiological characteristics is presented in Supplementary Figure S4. To test how virtual patients with different allelic variants of *CYP2C9* would respond to losartan treatment, we estimated the values of the *k*_*block*_ parameter, representing the AT1-receptor blocking activity of the drug, for different *CYP2C9* genotypes as described in Materials and Methods (Table 4). Each of the 100 virtual patients was simulated with each of the six values of *k*_*block*_, i.e., the difference between the groups for each genotype consisted only in *CYP2C9* activity.

After simulating the treatment of virtual patients with the found values of *k*_*block*_, we compared our results with clinical study by Sinitsina et al. (2023), where authors had investigated the antihypertensive effect of losartan monotherapy in hypertensive patients with different *CYP2C9* genotypes. In this study, patients were divided into two groups: 55 patients with the CYP2C9*1/CYP2C9*1 genotype and 26 patients carrying CYP2C9*2 or CYP2C9*3 variant alleles (13 patients with the CYP2C9*1/CYP2C9*2 genotype, 9 patients with the CYP2C9*1/CYP2C9*3 genotype, 2 patients with the CYP2C9*2/CYP2C9*2 genotype, and 2 patients with the CYP2C9*2/CYP2C9*3 genotype). Patients with the CYP2C9*3/CYP2C9*3 genotypes were not included in the study due to the rarity of this genotype.

**FIGURE 6:**
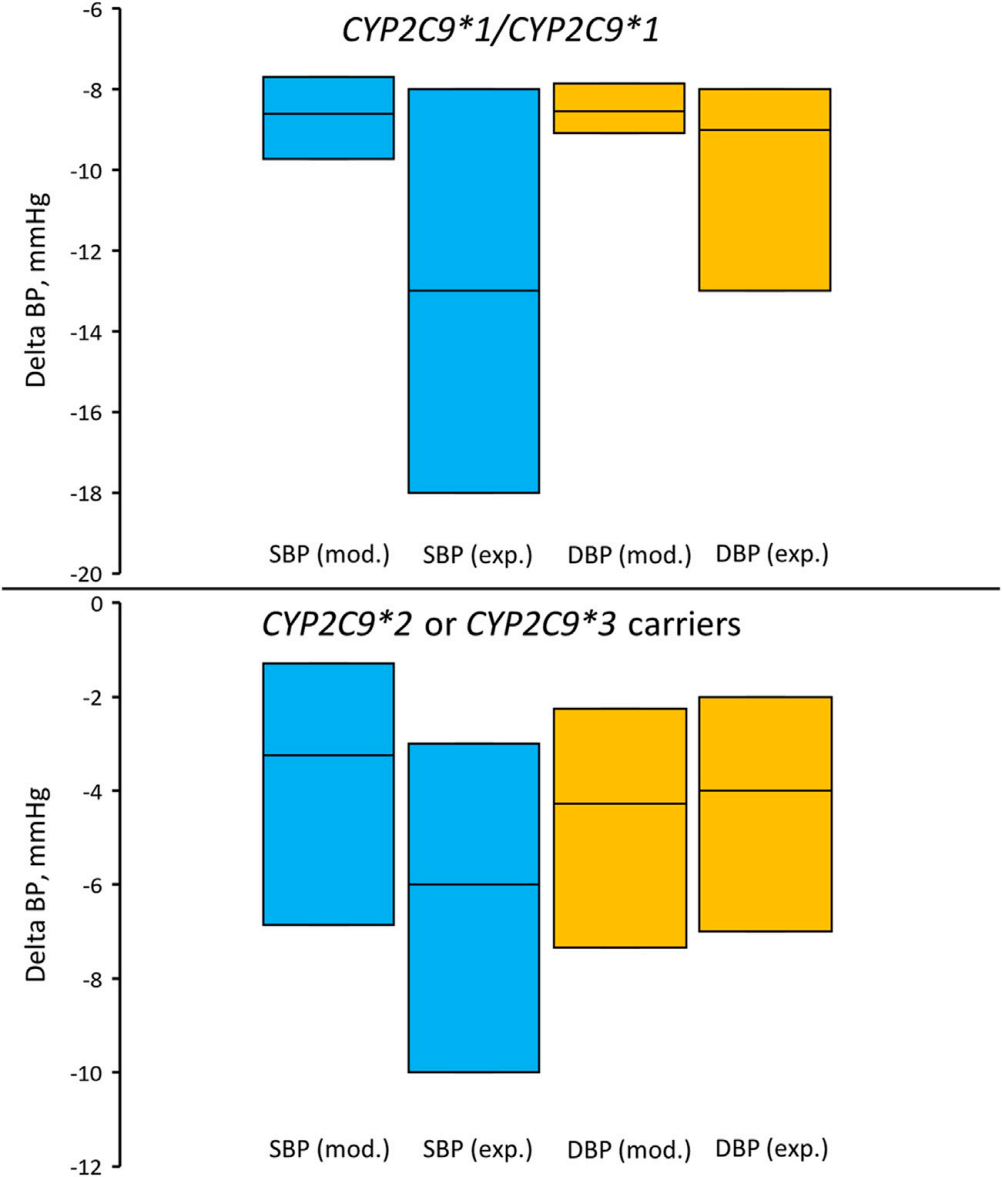
Comparison of simulated and experimentally obtained (Sinitsina et al., 2023) systolic and diastolic blood pressure responses to losartan treatment in patients with the CYP2C9*1/CYP2C9*1 genotype (top) and in patients who are carriers of CYP2C9*2 or CYP2C9*3 (bottom). Box plots show median and interquartile ranges. Delta BP, blood pressure response; SBP (mod.), simulated systolic blood pressure; DBP (mod.), simulated diastolic blood pressure; SBP (exp.), experimentally obtained systolic blood pressure; DBP (exp.), experimentally obtained diastolic blood pressure. In the experimental study, 55 patients were included in the CYP2C9*1/CYP2C9*1 group and 26 patients in the group of carriers of CYP2C9*2 or CYP2C9*3 (13 patients with the CYP2C9*1/CYP2C9*2 genotype, 9 patients with the CYP2C9*1/CYP2C9*3 genotype, 2 patients with the CYP2C9*2/CYP2C9*2 genotype, and 2 patients with the CYP2C9*2/CYP2C9*3 genotype).

Similar to the clinical study, we divided our virtual patients into the same two groups. For the CYP2C9*1/CYP2C9*1 group, we used all 100 patients with the corresponding *k*_*block*_ value (0.886), while for the second group, we used the same patients but with *k*_*block*_ values of other genotypes. Сomparison of the simulation and experimental results is presented in Table 7 and Figure 6. Unfortunately, we were unable to statistically evaluate the difference between our simulations and the clinical trial results because the experimental study (Sinitsina et al., 2023) only presented data as median and interquartile range. Overall, the agreement between modeled and experimental blood pressure responses to losartan treatment was better in patients with CYP2C9*2 or CYP2C9*3 alleles than in CYP2C9*1/CYP2C9*1 patients.

**TABLE 7:** Comparison of blood pressure response to losartan treatment between the clinical trial by Sinitsina et al. (2023) and the model simulation.

Group of patients	Blood pressure	Experimental data, mmHg (median (Q1; Q3))	Model simulation, mmHg (median (Q1; Q3))
CYP2C9*1/CYP2C9*1	Systolic	−13 (−8; −18)	−8.602 (−7.692; −9.734)
	Diastolic	−9 (−8; −13)	−8.537 (−7.855; −9.085)
CYP2C9*2 or CYP2C9*3 carriers	Systolic	−6 (−3; −10)	−3.238 (−1.286; −6.856)
	Diastolic	−4 (−2; −7)	−4.275 (−2.249; −7.347)

Q1, first quartile; Q3, third quartile.

In experimental study (Sinitsina et al., 2023), 55 patients were included in the CYP2C9*1/CYP2C9*1 group and 26 patients in the group of carriers of CYP2C9*2 or CYP2C9*3 (13 patients with the CYP2C9*1/CYP2C9*2 genotype, 9 patients with the CYP2C9*1/CYP2C9*3 genotype, 2 patients with the CYP2C9*2/CYP2C9*2 genotype, and 2 patients with the CYP2C9*2/CYP2C9*3 genotype).

**FIGURE 7:**
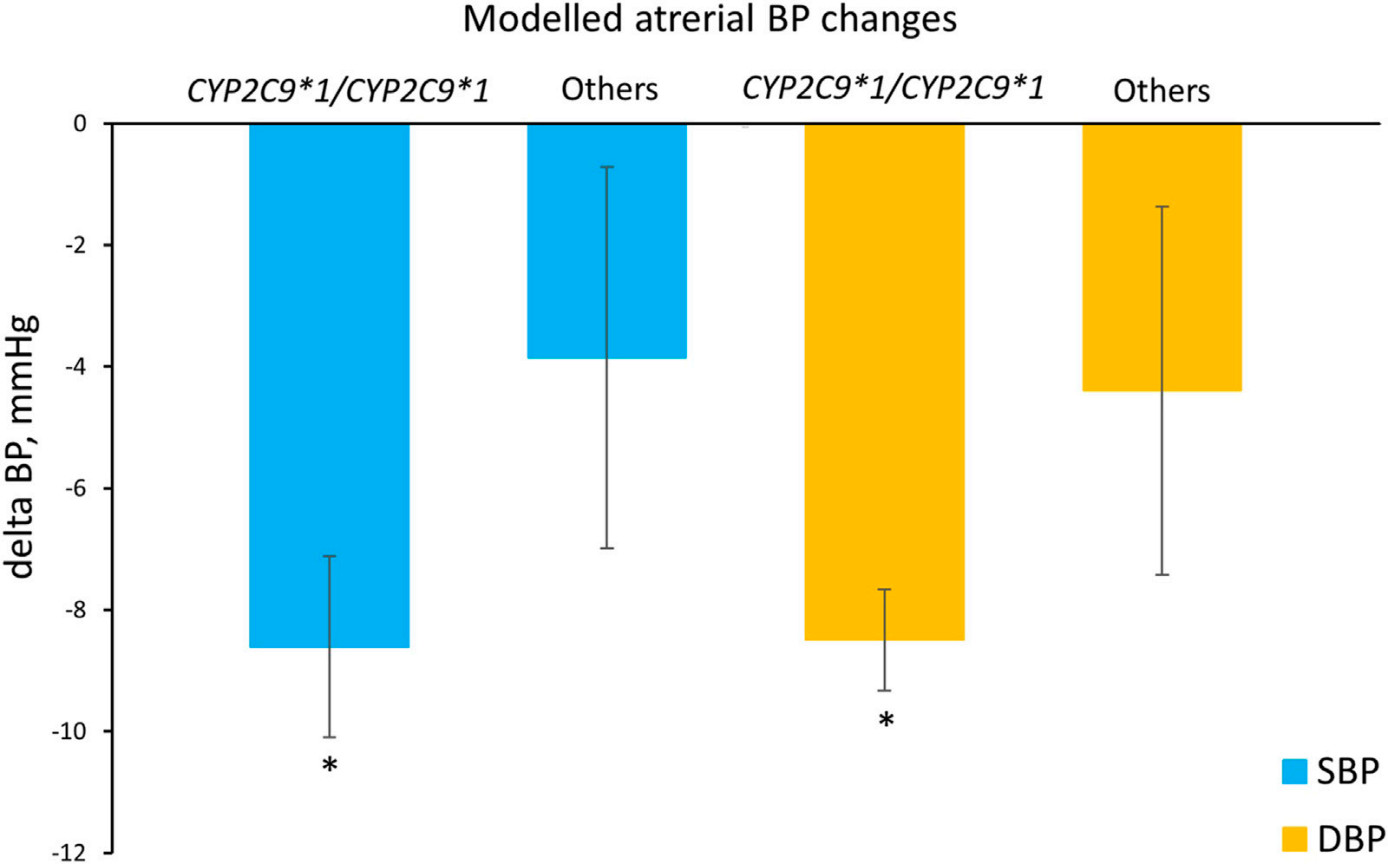
Comparison of simulated systolic and diastolic blood pressure responses to losartan monotherapy in patients with the CYP2C9*1/CYP2C9*1 genotype and in patients who are carriers of CYP2C9*2 or CYP2C9*3 (others). Values are given as mean ± SD. Delta BP, blood pressure response; SBP, systolic blood pressure; DBP, diastolic blood pressure. *P-value < 0.0001 vs. others. Comparisons were performed by unpaired Student’s t-test.

In addition, we compared the simulated systolic and diastolic blood pressure responses to losartan therapy between patients with the CYP2C9*1/CYP2C9*1 genotype and CYP2C9*2 or CYP2C9*3 carriers (Figure 7). As a result, we can conclude that virtual patients with the CYP2C9*1/CYP2C9*1 genotype had a greater reduction in both systolic and diastolic blood pressure after treatment with losartan, than patients with CYP2C9*2 or CYP2C9*3 alleles (*p*-value < 0.0001).

**FIGURE 8:**
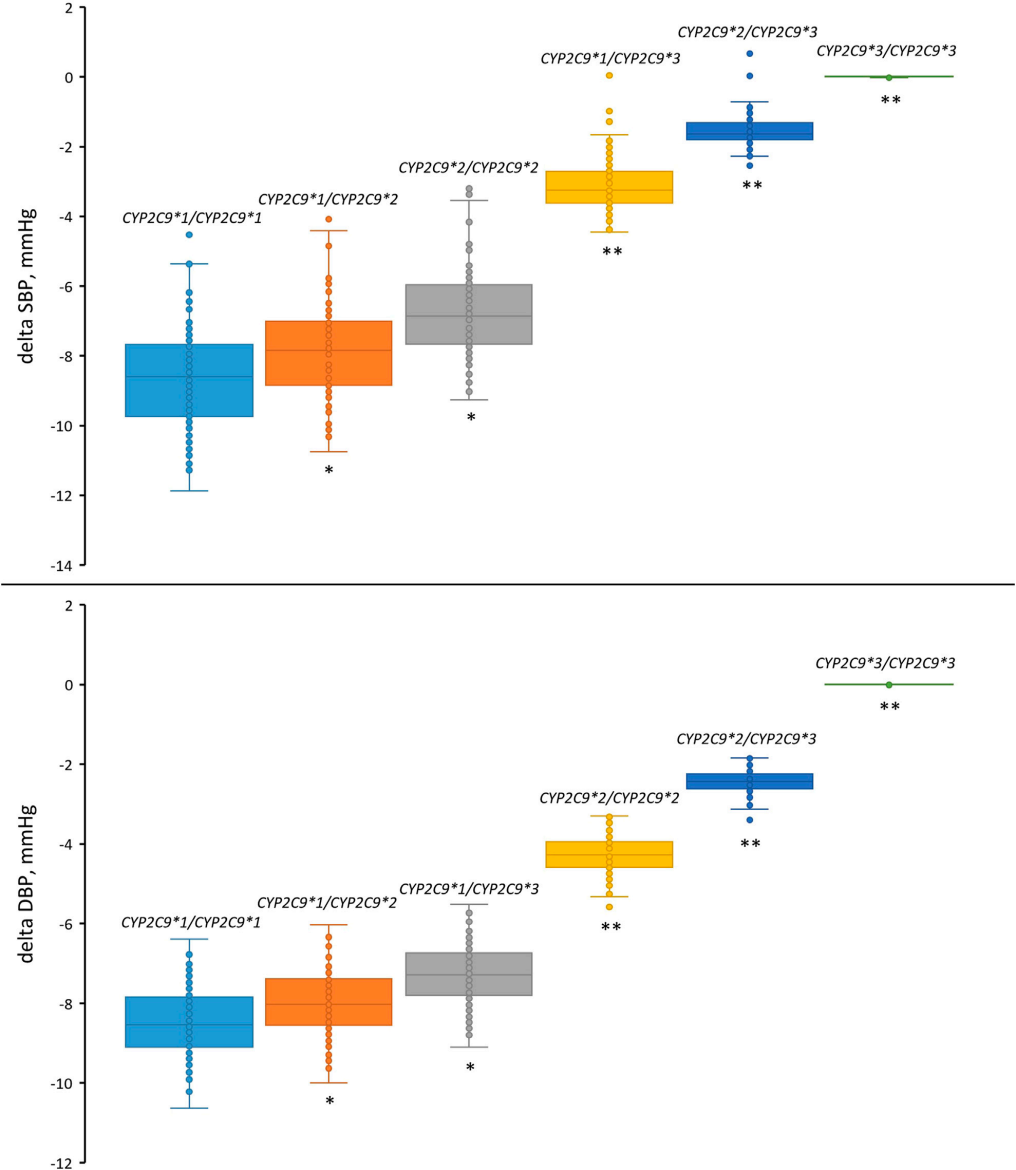
Comparison of simulated systolic and diastolic blood pressure responses to losartan monotherapy in patients with different *CYP2C9* genotypes. Box plots show medians, interquartile ranges, maximum, and minimum. Delta SBP, systolic blood pressure response; Delta DBP, diastolic blood pressure response. *P-value < 1E-03 vs. CYP2C9*1/CYP2C9*1. **P-value < 1E-10 vs. CYP2C9*1/CYP2C9*1. Comparisons were performed by unpaired Student’s t-test using the Bonferroni cutoff for significance (*P*-value < 0.0083, i.e., 0.05/number of *CYP2C9* genotypes).

We then examined the antihypertensive effect of losartan across all *CYP2C9* genotypes (Figure 8; Table 8). As can be seen, patients carrying the CYP2C9*2 allele had a similar reduction in blood pressure as patients with the CYP2C9*1/CYP2C9*1 genotype. At the same time, patients with the CYP2C9*3 allele had a significantly lower blood pressure response to losartan than patients with the wild-type genotype, while patients with CYP2C9*3/CYP2C9*3 did not experience any effect from losartan treatment.

**TABLE 8:** Simulated blood pressure response to losartan across *CYP2C9* genotypes.

Genotype	Systolic blood pressure		Diastolic blood pressure	
	Response (mean ± SD)	*P*-value	Response (mean ± SD)	*P*-value
CYP2C9*1/CYP2C9*1	−8.610 ± 1.488	-	−8.498 ± 0.834	-
CYP2C9*1/CYP2C9*2	−7.820 ± 1.379	1.34E−04*	−7.991 ± 0.793	3.31E−04*
CYP2C9*2/CYP2C9*2	−6.763 ± 1.240	1.34E−04*	−7.274 ± 0.736	3.31E−04*
CYP2C9*1/CYP2C9*3	−3.142 ± 0.758	1.86E−69**	−4.277 ± 0.480	7.70E−54**
CYP2C9*2/CYP2C9*3	−1.543 ± 0.463	1.57E−76**	−2.442 ± 0.301	6.55E−67**
CYP2C9*3/CYP2C9*3	0.007 ± 0.009	3.53E−78**	0.002 ± 0.003	3.73E−78**

^*^ *P*-value < 1E-03 vs. CYP2C9*1/CYP2C9*1.

^**^ *P*-value < 1E-10 vs. CYP2C9*1/CYP2C9*1.

Comparisons were performed by unpaired Student’s t-test using the Bonferroni cutoff for significance (*P*-value < 0.0083, i.e., 0.05/number of CYP2C9 genotypes).

## 4 Discussion

The genetic factors are known to be able to contribute to an increase in blood pressure by 30%–50% (Dominiczak et al., 2000; Menni et al., 2013), and the highly polymorphic *CYP2C9* gene plays a critical role in the metabolism of the antihypertensive drug losartan (Sullivan-Klose et al., 1996). Therefore, we integrated the most frequent *CYP2C9* genotypes into the model to accurately predict the plasma profiles of losartan and E-3174 in patients with the corresponding genotypes.

The aim of this study was to modify an existing mathematical model that describes the distribution of losartan and its metabolite E-3174 (Karatza and Karalis, 2020). The final model was expected to predict plasma concentration-time curves of both compounds for patients with the most common alleles of the *CYP2C9* gene: CYP2C9*1, CYP2C9*2, and CYP2C9*3.

Firstly, we reproduced the original model in the BioUML software and then redefined the model parameter values using the stochastic ranking evolutionary strategy optimization method. Secondly, we performed the identifiability analysis of the model parameters with the redefined values. In addition, we calculated the pharmacokinetic parameters (C_max_, t_max_, t_1/2_, AUC and AUC_ratio_) for the curves predicted by the model and then compared these values with clinical data to verify the model. Finally, we assessed the effect of losartan treatment in virtual hypertensive patients with different *CYP2C9* genotypes using the cardiorenal model (Kutumova et al., 2022; Kutumova et al., 2021).

The model showed good agreement with clinical data. However, some discrepancies were observed between simulated and clinical data for some pharmacokinetic characteristics across *CYP2C9* genotypes. For example, a discrepancy between predicted and experimental values was observed for t_1/2, E-3174_ (terminal elimination half-life of E-3174). A possible explanation for this fact is that the experimental study did not indicate which time points of the linear part of the semilogarithmic plasma concentration-time curve were used to calculate the terminal elimination half-life (t_1/2_) (Yasar et al., 2002). For losartan this is probably not as important, since it reaches peak concentrations quickly (within an hour) and then declines steadily (Supplementary Figure S5, top). Consequently, the semilogarithmic concentration-time curve of losartan rapidly becomes linear after drug administration (Supplementary Figure S6, top). On the other hand, for E-3174 we can observe a different form of concentration-time profile: E-3174 reaches its maximum concentration later than losartan, approximately 3–4 h after drug administration (Supplementary Figure S5, bottom). Therefore, on a semilogarithmic plot, the profile of E-3174 is flatter (Supplementary Figure S6, bottom) than that of losartan. For this reason, we think that the choice of time points used to calculate t_1/2_ is more crucial for E-3174 than for losartan.

There may also be other reasons for the observed discrepancies: 1) insufficient number of patients with certain genotypes in the clinical trial (Yasar et al., 2002) due to low allele frequencies of CYP2C9*2 and CYP2C9*3, 2) small number of experimental concentration-time points used for model training, and 3) high inter-subject variability in the plasma concentration-time curves of losartan and E-3174. It is known that losartan belongs to the first class of drugs according to the BCS (Biopharmaceutics Classification System), possessing high solubility and permeability (Chavda et al., 2010). Emptying of gastric contents into the duodenum through the pyloric valve is the rate-limiting process for absorption of these drugs (Sugihara et al., 2015; Tsume and Amidon, 2010). Thus, the high inter-patient variability in losartan and E-3174 plasma profiles may be due to physiological differences in gastric emptying processes. In addition, individuals may experience different phases of the migrating motor complex cycle (the regular pattern of gastric motility during fasting) while taking medications (Amidon et al., 1991; Higaki et al., 2008; Langguth et al., 1994; Oberle and Amidon, 1987; Takamatsu et al., 2002). Besides, discrepancies may arise because not only various *CYP2C9* alleles have different impact on the pharmacokinetics of losartan, but polymorphisms in other genes can also affect the absorption, distribution, metabolism, or excretion of this drug. For example, different alleles of the *ABCB1* (ATP-binding cassette, subfamily B, number 1) gene are associated with different rates of early-phase oral absorption of losartan (Shin et al., 2020).

Modeling the antihypertensive effect of losartan therapy in a group of virtual patients with different *CYP2C9* genotypes yielded the expected result. Individuals with the CYP2C9*1/CYP2C9*1 (wild-type) genotype responded better to losartan therapy than patients with CYP2C9*2 or CYP2C9*3 alleles. However, the simulated blood pressure responses were slightly different from those obtained in the clinical study (Sinitsina et al., 2023) (Table 7; Figure 6). This is probably due to the fact that we used the *k*_*block*_ value (0.886) from the study by Kutumova et al. (2022), where it was estimated based on data from another clinical study (Porthan et al., 2009). A second possible reason for this discrepancy is that in the study by Sinitsina et al. (2023), the losartan dose for patients was altered during the study period, while we used *k*_*block*_ = 0.886, adjusted for a constant oral dose of losartan 50 mg.

### 4.1 Limitations of the study

The main limitation of this study is related to the small number of patients with different *CYP2C9* genotypes whose clinical data were used for model validation and verification. This is because the frequencies of the variant alleles CYP2C9*2 and CYP2C9*3 are also very low.

### 4.2 Future prospects

Accumulating the results on modeling the influence of various genetic factors on the regulation of arterial pressure may further enable studying their cross-influence on the development and treatment of arterial hypertension. In particular, other genes that could influence the absorption, distribution, metabolism, and excretion of losartan can be considered, which will allow for more accurate prediction of the pharmacokinetics and pharmacodynamics of losartan.

## Data Availability

The implementation of the model is available in the web version of the BioUML (https://sirius-web.org/bioumlweb/#de=data/Collaboration%20(git)/CYP2C9_losartan_metabolism/Data/Diagrams/Babaev2024%20-%20CYP2C9%20variants) platform and in the BioModels database (Malik-Sheriff et al., 2020) with ID MODEL2412180002 (https://www.ebi.ac.uk/biomodels/MODEL2412180002). Also, the description of the study, the models in SBML format, and instructions on how to reproduce the results are stored in the GitHub (https://github.com/DBgentech2023sirius/CYP2C9) and GitLab (https://gitlab.sirius-web.org/virtual-patient/CYP2C9_losartan_metabolism) repositories. Initial model (Karatza and Karalis, 2020) is also available in the BioModels database with ID MODEL2412180001 (https://www.ebi.ac.uk/biomodels/MODEL2412180001).

## Footnotes

1
https://www.ncbi.nlm.nih.gov/protein/NP_000762.2

2
https://www.pharmvar.org/gene/CYP2C9

3
https://gnomad.broadinstitute.org

4
http://www.biouml.org/

5
https://www.ebi.ac.uk/biomodels/MODEL2202160001

6
https://plotdigitizer.com/app
