# Supplementary material for: Mathematical modeling of pharmacokinetics and pharmacodynamics of losartan in relation to CYP2C9 allele variants
Source: Front Syst Biol. 2025 May 21;5:1504077. doi: 10.3389/fsysb.2025.1504077 (PMC12342004; doi:10.3389/fsysb.2025.1504077)
Supplement: Supplementary file 1 [file Supplementaryfile1.docx]

# Supplementary Material

### **Supplementary Table S1.** Model equations

| The number of reaction in Figure 2 | Equation | A physiological process in the body described by the equation |
| --- | --- | --- |
| 1 | *max(0, a*sin(2*π/b*time))*[L_stm_]* | Open-close cycles of the gastric pyloric valve |
| 2 | *k_a_*[L_int_]* | Losartan absorption from the small intestine into the blood |
| 3 | *k_m_*[L_cc_]*start_CYP2C9* | Conversion of losartan to E-3174 by CYP2C9 |
| 4 | *k_elm_*[C_cc_]* | E-3174 elimination from the body |
| 5 | *k_elp_*[L_cc_]* | Losartan elimination from the body |
| 6 | *k_12_*[L_cc_]* | Losartan transfer from the blood to other organs and tissues |
| 7 | *k_21_*[L_pc_]* | Losartan transfer from organs and tissues into the blood |

### **Supplementary Table S2.** Model parameters and variables

| Parameter/variable | Definition |
| --- | --- |
| *a* | The amplitude of the sinusoidal equation, which describes open-close cycles of the gastric pyloric valve (h^-1^) |
| *b* | The period of the sinusoidal equation, which describes open-close cycles of the gastric pyloric valve (h) |
| *k_a_* | The rate constant of the losartan absorption from the small intestine into the blood (h^-1^) |
| *k_m_* | The rate constant of the conversion of losartan to E-3174 by CYP2C9 (h^-1^) |
| *k_elm_* | The E-3174 elimination rate constant (h^-1^) |
| *k_elp_* | The losartan elimination rate constant (h^-1^) |
| *k_12_* | The rate constant of losartan transfer from the central to the peripheral compartment (h^-1^) |
| *k_21_* | The rate constant of losartan transfer from the peripheral to the central compartment (h^-1^) |
| *[L_stm_]* | The amount of losartan in the stomach (pmol) |
| *[L_int_]* | The amount of losartan in the small intestine (pmol) |
| *[L_cc_]* | The amount of losartan in the central compartment (pmol) |
| *[L_pc_]* | The amount of losartan in the peripheral compartment (pmol) |
| *[C_cc_]* | The amount of E-3174 in the central compartment (pmol) |
| *start_CYP2C9* | The discrete variable used to describe the delay in conversion of losartan to E-3174, which can take the values 0 (no CYP2C9 activity) or 1 (presence of CYP2C9 activity) |

### **Supplementary Table S3.** Equations, discrete events and initial assignments of the model

| Equation/event/initial assignment | Definition |
| --- | --- |
| *When time > T:*  *start_CYP2C9 = 1* | The discrete event used to describe the delay in the conversion of losartan to E-3174 for time T |
| *[L_stm_](0) = 0.05/461.01*10^12^* | The initial assignment that determines the 50 mg oral dose of losartan potassium (pmol): 0.05 g = 50 mg, 461.01 g/mol - molecular weight of losartan potassium, 10^12^ - coefficient to convert moles to pmoles |
| *k_elp_ = CL_p_/V_p1_* | The algebraic equation that determines the value of k_elp_ |
| *k_elm_ = CL_m_/V_m_* | The algebraic equation that determines the value of k_elm_ |
| *k_12_ = Q/V_p1_* | The algebraic equation that determines the value of k_12_ |
| *k_21_ = Q/V_p2_* | The algebraic equation that determines the value of k_21_ |
| *C_p_ = [L_cc_]/(V_p1_*1000)* | The algebraic equation that determines the value of C_p_ |
| *C_m_ = [C_cc_]/(V_m_*1000)* | The algebraic equation that determines the value of C_m_ |

### **Supplementary Table S4.** Model parameters

| Variable | Definition |
| --- | --- |
| *T* | The delay time of losartan conversion to E-3174 (h) |
| *[L_stm_](0)* | The amount of losartan in the stomach at the beginning of the model simulation (pmol) |
| *CL_p_* | Apparent clearance of losartan (L/h) |
| *CL_m_* | Apparent clearance of E-3174 (L/h) |
| *V_p1_* | Apparent volume of distribution of losartan in the central compartment (L) |
| *V_p2_* | Apparent volume of distribution of losartan in the peripheral compartment (L) |
| *V_m_* | Apparent volume of distribution of E-3174 in the central compartment (L) |
| *Q* | Apparent inter-compartmental (central-peripheral) clearance of losartan (L/h) |
| *C_p_* | Concentration of losartan in the central compartment (nM) |
| *C_m_* | Concentration of E-3174 in the central compartment (nM) |

### **Supplementary Table S5.** Experimental points used to optimize the E-max model

| Losartan oral dose (mg) | AUC_E-3174_ (nmol*h/L) | *k_block_* (unitless) |
| --- | --- | --- |
| 25 | 1998.318 | 0.1 |
| 50 | 3996.635 | 0.886 |
| 100 | 7993.271 | 0.954 |

### **Supplementary Table S6.** Fitted values of the E-max model coefficients

| Coefficient | Value |
| --- | --- |
| ED_50_ (nmol*h/L) | 2741.565 |
| E_max_ (unitless) | 0.955 |
| ɑ (unitless) | 6.785 |

### **Supplementary Table S7.** Digitized concentration-time data for *CYP2C9*1/CYP2C9*1*, *CYP2C9*2/CYP2C9*2*, and *CYP2C9*3/CYP2C9*3* genotypes

| *CYP2C9*1/CYP2C9*1* | | | *CYP2C9*2/CYP2C9*2* | | | *CYP2C9*3/CYP2C9*3* | | |
| --- | --- | --- | --- | --- | --- | --- | --- | --- |
| Time (h) | Losartan (nM) | E-3174 (nM) | Time (h) | Losartan (nM) | E-3174 (nM) | Time (h) | Losartan (nM) | E-3174 (nM) |
| 0.35 | 337.17 | 10.93 | 0.61 | 713.00 | 48.80 | 0.39 | 22.06 | - |
| 0.86 | 675.00 | 57.26 | 0.98 | 338.05 | 159.55 | 0.88 | 706.00 | - |
| 1.32 | 315.35 | 158.84 | 1.59 | 272.13 | 201.53 | 1.46 | 633.70 | - |
| 1.82 | 258.00 | 280.51 | 3.74 | 238.13 | 441.50 | 1.90 | 457.49 | 5.72 |
| 3.85 | 88.46 | 603.00 | 5.79 | 211.88 | 486.00 | 3.90 | 234.58 | 25.00 |
| 5.87 | 54.46 | 331.57 | 7.80 | 92.00 | 343.74 | 5.95 | 180.75 | 18.47 |
| 7.89 | 46.85 | 221.95 | 10.05 | 54.84 | 204.92 | 7.90 | 116.42 | 16.48 |
| 9.87 | 23.60 | 158.84 | 12.06 | 21.54 | 118.16 | 9.95 | 76.22 | 16.21 |
| 11.84 | - | 102.83 | - | - | - | 11.95 | - | 13.12 |
| 23.94 | - | 16.33 | - | - | - | - | - | - |

**Supplementary Table S8**. Optimized values of the model parameters

###

| Model parameter | Initial value | Fitted value |
| --- | --- | --- |
| *CL_m_* (L/h) | 5.500 | 16.456 |
| *CL_p_* (L/h) | 184.000 | 30.401 |
| *T* (h) | 0.271 | 0.819 |
| *V_m_* (L) | 9.660 | 57.598 |
| *V_p1_* (L) | 43.700 | 56.175 |
| *V_p2_* (L) | 1160.000 | 135.63 |
| *Q* (L/h) | 129.000 | 217.103 |
| *a* (h^-1^) | 4.560 | 6.645 |
| *k_a_* (h^-1^) | 1.930 | 5.127 |

#####

####

####

####

####

####

####

####

####

####

####

####

####

####

####

####

#### **
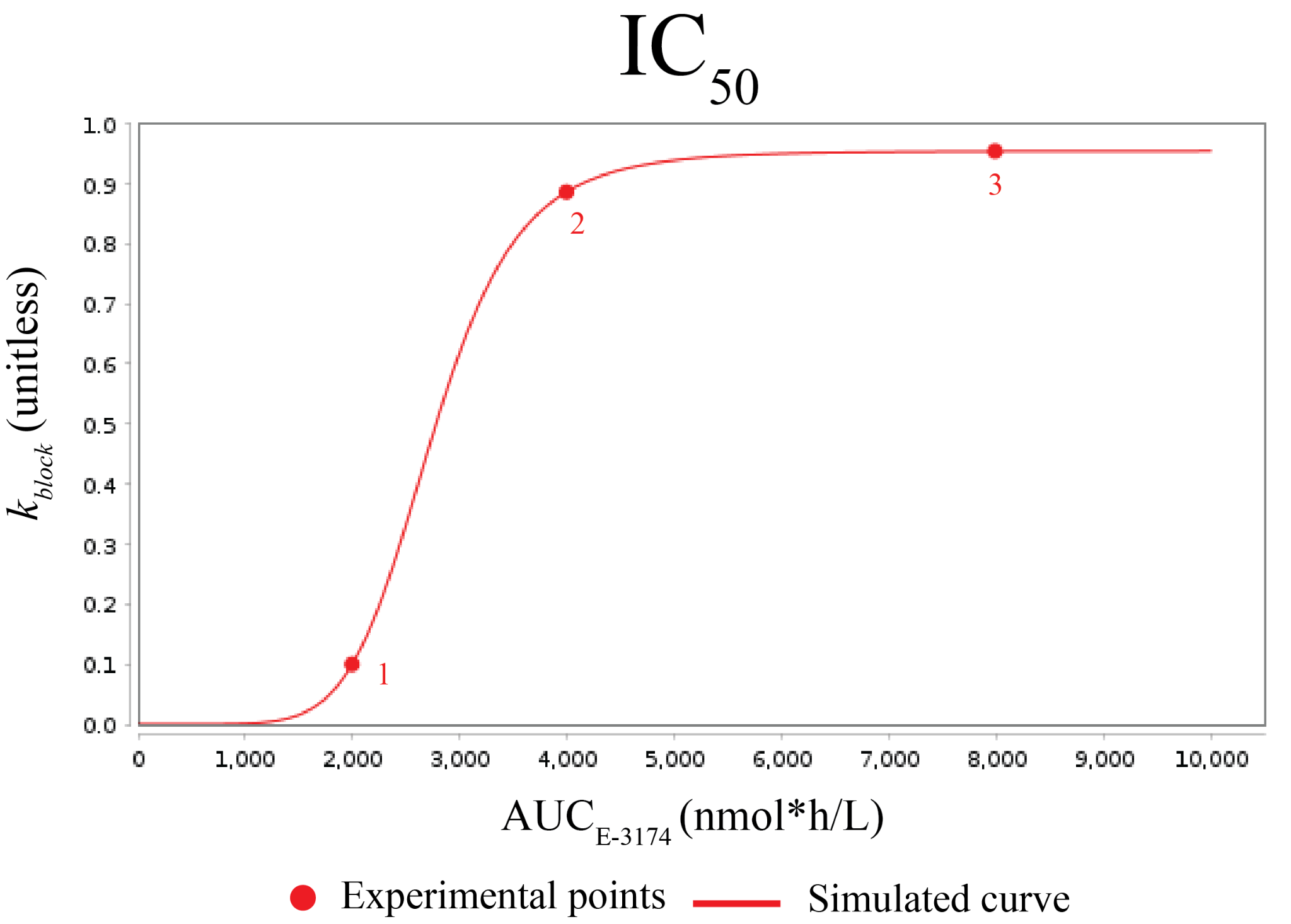
**

#### **Supplementary Figure S1**. IC_50_ plot of losartan; experimental points correspond to oral doses of losartan of 25 mg (point 1), 50 mg (point 2), and 100 mg (point 3).


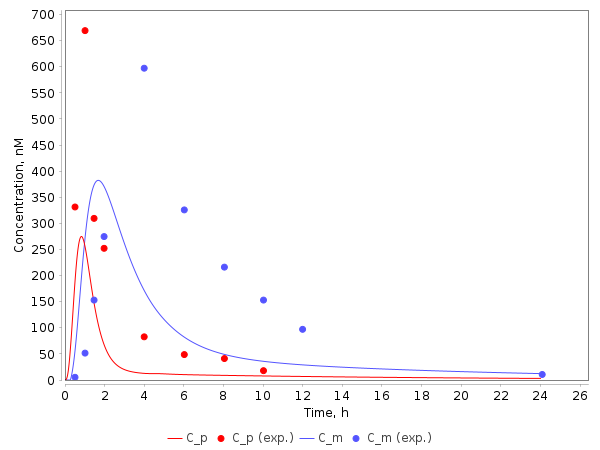


#### **Supplementary Figure S2**. Discrepancy between the predictions of the original model and experimental data [(Yasar et al., 2002)](https://www.zotero.org/google-docs/?UbwN7s) when changing the dosage of orally administered losartan potassium from 100 to 50 mg. To check the correspondence, experimental points obtained for the *CYP2C9*1/CYP2C9*1* genotype were chosen, since this genotype is typical for most people. C_p and C_m are the plasma concentrations of losartan and E-3174 predicted by the model, respectively, C_p (exp.) and C_m (exp.) are experimentally obtained plasma concentrations of losartan and E-3174, respectively.


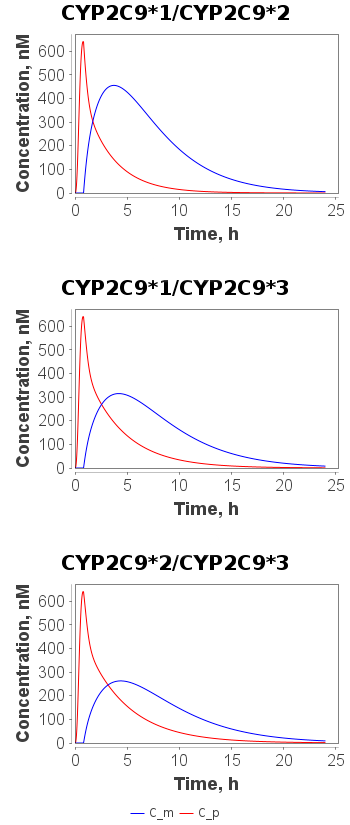


#### **Supplementary Figure S3**. Predicted losartan (C_p) and E-3174 (C_m) concentration-time curves for heterozygous *CYP2C9* genotypes.


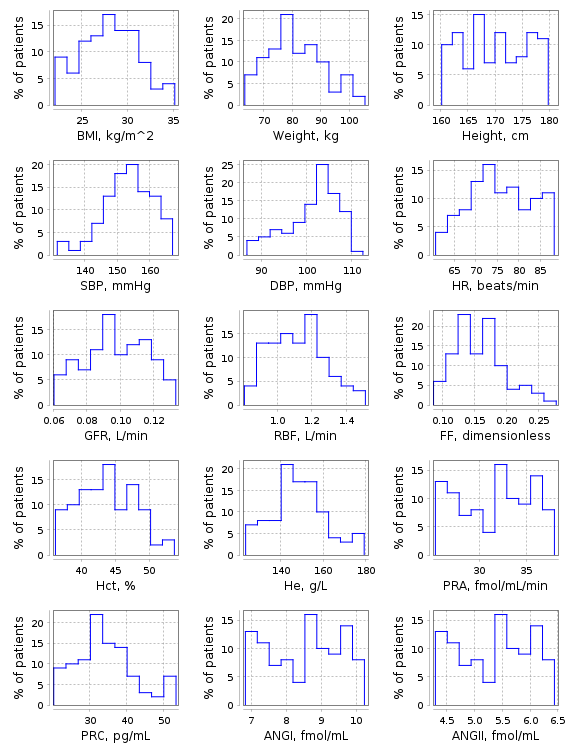


#### **Supplementary Figure S4.** Distribution of physiological characteristics of the virtual hypertensive population. BMI, body mass index; SBP, systolic blood pressure; DBP, diastolic blood pressure; HR, heart rate; GFR, glomerular filtration rate; RBF, renal blood flow; FF, filtration fraction; Hct, hematocrit; He, hemoglobin; PRA, plasma renin activity; PRC, plasma renin concentration; ANGI, plasma angiotensin I concentration; ANGII, plasma angiotensin II concentration.


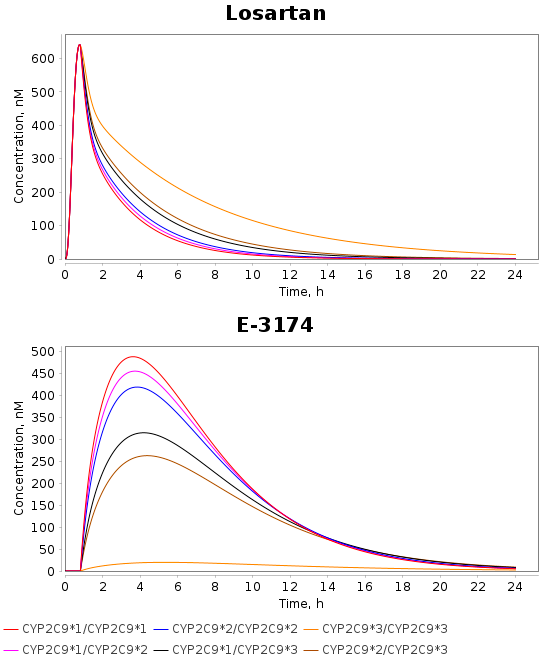


#### **Supplementary Figure S5**. Concentration-time profiles of losartan (top) and E-3174 (bottom) for all simulated *CYP2C9* genotypes.


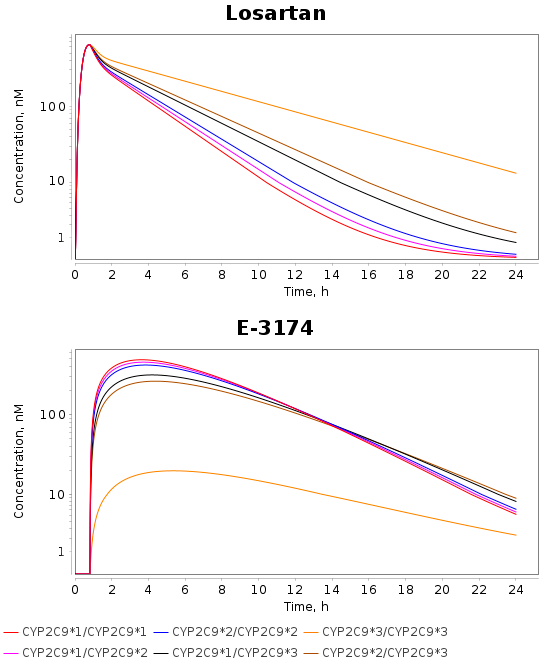


#### **Supplementary Figure S6**. Semilogarithmic сoncentration-time curves of losartan (top) and E-3174 (bottom) for all simulated *CYP2C9* genotypes.
